# Abacavir Drug Exposures in African Children Under 14 kg Using Pediatric Solid Fixed Dose Combinations According to World Health Organization Weight Bands

**DOI:** 10.1093/jpids/piad082

**Published:** 2023-10-05

**Authors:** Suthunya Chupradit, Dalton C Wamalwa, Elizabeth Maleche-Obimbo, Adeodata R Kekitiinwa, Juliet Mwanga-Amumpaire, Elizabeth A Bukusi, Winstone M Nyandiko, Joseph K Mbuthia, Alistair Swanson, DNDi Clinical Team, Tim R Cressey, Baralee Punyawudho, Victor Musiime

**Affiliations:** PhD’s Degree Program in Pharmacy, Faculty of Pharmacy, Chiang Mai University, Chiang Mai, Thailand; Department of Paediatrics and Child Health, University of Nairobi, Nairobi, Kenya; Department of Paediatrics and Child Health, University of Nairobi, Nairobi, Kenya; Baylor College of Medicine Children’s Foundation, Kampala, Uganda; Epicentre, Mbarara, Uganda; Centre for Microbiology Research, Kenya Medical Research Institute, Kisumu, Kenya; Department of Child Health and Paediatrics—Moi University, AMPATH and Moi Teaching and Referral Hospital, Eldoret, Kenya; Gertrude’s Children’s Hospital, Nairobi, Kenya; Drugs for Neglected Diseases Initiative, Geneva, Switzerland; Drugs for Neglected Diseases Initiative, Nairobi, Kenya; Drugs for Neglected Diseases Initiative, New York, USA; Drugs for Neglected Diseases Initiative, Geneva, Switzerland; Drugs for Neglected Diseases Initiative, Nairobi, Kenya; Drugs for Neglected Diseases Initiative, New York, USA; AMS/IRD Research Collaboration, Faculty of Associated Medical Sciences, Chiang Mai University, Chiang Mai, Thailand; Department of Molecular & Clinical Pharmacology, University of Liverpool, Liverpool, UK; Department of Pharmaceutical Care, Faculty of Pharmacy, Chiang Mai University, Chiang Mai, Thailand; Joint Clinical Research Centre, Kampala, Uganda; Department of Paediatrics and Child Health, Makerere University, Kampala, Uganda

**Keywords:** Abacavir, African children, NONMEM, dose optimization, population pharmacokinetics

## Abstract

**Background:**

The pharmacokinetics of abacavir (ABC) in African children living with HIV (CLHIV) weighing <14 kg and receiving pediatric fixed dose combinations (FDC) according to WHO weight bands dosing are limited. An ABC population pharmacokinetic model was developed to evaluate ABC exposure across different World Health Organization (WHO) weight bands.

**Methods:**

Children enrolled in the LIVING study in Kenya and Uganda receiving ABC/lamivudine (3TC) dispersible tablets (60/30 mg) according to WHO weight bands. A population approach was used to determine the pharmacokinetic parameters. Monte Carlo simulations were conducted using an in silico population with demographic characteristics associated with African CLHIV. ABC exposures (AUC_0–24_) of 6.4–50.4 mg h/L were used as targets.

**Results:**

Plasma samples were obtained from 387 children. A 1-compartment model with allometric scaling of clearance (CL/F) and volume of distribution (V/F) according to body weight best characterized the pharmacokinetic data of ABC. The maturation of ABC CL/F was characterized using a sigmoidal *E*_max_ model dependent on postnatal age (50% of adult CL/F reached by 0.48 years of age). Exposures to ABC were within the target range for children weighing 6.0–24.9 kg, but children weighing 3–5.9 kg were predicted to be overexposed.

**Conclusions:**

Lowering the ABC dosage to 30 mg twice daily or 60 mg once daily for children weighing 3–5.9 kg increased the proportion of children within the target and provided comparable exposures. Further clinical study is required to investigate clinical implications and safety of the proposed alternative ABC doses.

## BACKGROUND

Current World Health Organization (WHO) HIV treatment guidelines recommend abacavir (ABC), lamivudine (3TC), and dolutegravir (DTG) as the preferred first-line antiretroviral agents for children living with HIV (CLHIV) older than 4 weeks of age and weighing at least 3 kg [1]. Generic solid fixed dose combinations (FDC) of ABC/3TC are widely available in Sub-Saharan Africa. Pediatric ABC/3TC dispersible tablets are primarily used for young children and are dosed per WHO weight bands: 60, 90, 120, 150, and 180 mg of ABC twice daily for children weighing 3–5.9, 6–9.9, 10–13.9, 14–19.9, and 20–24.9 kg, respectively. A limitation of weight band dosing with FDC is the risk of over and under dosing within each weight band. The WHO weight band doses of ABC with the ABC/3TC FDC tablet can lead to significantly higher mg/kg doses than the approved 8 mg/kg dose of ABC with the liquid formulation, especially in the lowest weight band [1]. For example, a child weighing 4 kg would receive 60 mg of ABC twice daily (15 mg/kg) with the FDC dispersible tablet per WHO dosing, as opposed to 32 mg (8 mg/kg) of ABC. In addition, previous studies on the pharmacokinetics of ABC oral solution in very young infants indicated a significantly high exposure, and lower doses of ABC have been suggested [2, 3]. The current recommended ABC doses with the oral solution in neonates and infants <3 months of age are based on a recent pharmacokinetic modeling and simulation study of pediatric data targeting adult plasma ABC exposures using both exact (mg/kg) and WHO weight band doses [3]. To date, limited pharmacokinetic data are available in children less than 14 kg receiving the ABC/3TC dispersible tablet per WHO weight band dosing.

Body weight is a major factor influencing ABC pharmacokinetics [4–11], but physiological maturation processes can also play a crucial role in the first year of life. The effect of size and age on pharmacokinetic parameters can be evaluated with a population pharmacokinetic approach.

Our aim was to develop a population pharmacokinetic model of ABC that includes African infants using the generic dispersible ABC/3TC tablet and to assess the drug exposures achieved using WHO weight band dosing recommendations, with a focus on children weighing between 3 and <14 kg.

## METHODS

### Study Population and Blood Collection

Data from children enrolled in the “Lopinavir based ART for HIV-Infected children Globally (LIVING)” study (ClinicalTrials.gov Identifier NCT02346487) were included in this analysis. Briefly, the LIVING study was conducted in Kenya and Uganda to evaluate the safety, effectiveness, pharmacokinetics, and acceptability of lopnavir/ritonavir (LPV/r) 40/10 mg oral pellets-based antiretroviral therapy (ART) in infants and young children with HIV who are unable to swallow tablets. The CLHIV who had never taken antiretrovirals (ARVs), were already on first-line liquid LPV/r-based therapy or had failed first line non-nucleoside reverse transcriptase inhibitor (NNRTI)-based therapy and weighed between 3 and 25 kg at the time of enrollment were included. An nucleoside reverse transcriptase inhibitor (NRTI) back-bone of pediatric ABC/3TC 60/30 mg or zidovudine (AZT)/3TC 60/30 mg dispersible tablets was administered with the LPV/r pellets depending on what was considered best for the child by the attending physician. Only children who received ABC/3TC dispersible tablets were included in this analysis. Children were ineligible for the study if they were planning to take or were already taking NNRTIs, entry inhibitors, integrase inhibitors, or protease inhibitors (PIs) other than LPV/r, if they had previously failed PI treatment and either had the presence or high suspicion of a PI resistance mutation, if they had a clinical condition requiring a prohibited medication, or if they had been treated with experimental drugs within 30 days prior to enrollment.

ABC was administered using ABC/3TC dispersible tablets (60/30 mg tablet) twice daily according to WHO weight band dosing: 1, 1.5, 2, 2.5, and 3 tablets for children 3–5.9, 6–9.9, 10–13.9, 14–19.9, and 20–24.9 kg, respectively. During study follow-up, sparse blood samples were collected at 1 month after enrollment in the study, and then every 6 months afterward. At 1 month, 3 blood samples were collected: immediately before the morning dose, 0–1 hour after taking the drug, and 4–8 hours after taking the drug. Two blood samples were collected every 6 months, 1 before the morning dose, and another between 2 and 6 hours after drug administration. Drug doses, timing of blood sampling, body weight, postnatal age (PNA), malnutrition status, and ARV regimens were recorded at each visit. This study was approved by the Ethics Committee of Pharmacy, Faculty of Pharmacy, Chiang Mai University (Chiang Mai, Thailand). The LIVING study was approved by ethical and regulatory bodies in Kenya and Uganda, following the respective country research guidelines.

### Determination of ABC Plasma Concentrations

Blood samples were processed within 1 hour and plasma were stored at −70°C until analysis. The samples were sent to a centralized laboratory for analysis, following an approved material transfer agreement by the in country ethical and regulatory bodies. Plasma ABC concentrations were measured using a validated liquid chromatography triple quadrupole mass spectrometer (LC-MS/MS) assay. All samples were analyzed at the Bio-analytical testing laboratory, Clinical Service Center, Faculty of Associated Medical Science, Chiang Mai University. The standard calibration curve was linear over a plasma concentration range of 20–2500 ng/mL. The lower limit of quantification (LLOQ) was 20 ng/mL. Intraday and interday variability (%CV) range were 0.6%–3.4% and 1.7%–3.0%, respectively. This laboratory participates in the USA NIH Clinical Pharmacology Quality Control program [12].

### Population Pharmacokinetic Analysis

The steady-state pharmacokinetics of ABC were analyzed with NONMEM® (version 7.3, Icon Development Solutions, Ellicott City, MD, USA) and Pirana (version 2.9.6) using a nonlinear mixed-effect modeling approach. The R software (version 3.1.2, http://www.r-project.org), ggplot2 package, and Xpose programs (version 4.5.0) were used for graphical analysis. The first-order conditional estimation with interaction (FOCEI) method was used to estimate pharmacokinetic parameters and their variabilities. The M3 method was also assessed to handle ABC concentrations below the quantification limit (BQL) using the Laplacian approximation method.

One- and 2-compartment models with first-order absorption with and without absorption lag time and first-order elimination were tested to describe ABC concentrations over time. Oral clearance (CL/F) and apparent volume of distribution (V/F) were scaled allometrically to account for the influence of body weight, with exponents fixed at 0.75 and 1, respectively. The influence of age on CL/F was evaluated using a sigmoidal maximum enzyme induction (*E*_max_) model to describe the enzyme maturation effect [13]:


$$\begin{array}{l} MF_{PNA}=\frac{PNA^{HILL}}{MA{T_{50}}^{HILL}+PNA^{HILL}} \end{array}$$ (1)

where MF is maturation effect, PNA is postnatal age in years and MAT_50_ is the PNA where the clearance is 50% of the mature clearance value. HILL is a coefficient that describes the steepness of the maturation-age correlation.

The interindividual variability (IIV) and interoccasion variability (IOV) were described using an exponential model. The residual unexplained variability was described by a proportional error model. The structural model was selected based on several factors, including the objective function value (OFV) changes, goodness-of-fit (GOF) plots, the precision of parameter estimates, and achievement of successful convergence. Once the appropriate structural model was obtained, the influence of potential covariates was evaluated. These covariates included gender, height, and malnutrition status, as determined by the weight-for-age *z*-score (WAZ), which is classified as normal (WAZ > −2), moderate (−3 < WAZ < −2), and severe (WAZ < −3); and were investigated using a stepwise approach. Continuous covariates were investigated using linear, exponential, and power models, while categorical covariates were investigated using additive, exponential, and fractional models. The selection of hierarchical models was based on specific criteria, with a decrease in OFV of 3.84 (*P* < .05) for forward selection and an increase of 6.63 (*P* < .01) for backward selection.

### Model Evaluation

The final model’s predictability and reliability were evaluated using prediction-corrected visual predictive checks (pcVPC) and bootstrapping [14, 15]. For pcVPC analysis, 1000 simulations were performed. The 95% confidence intervals (CIs) of the medians, 5th, and 95th percentiles of the simulated concentrations were plotted against the corresponding values of the observed data. For the bootstrap analysis, 1000 replicates were randomly selected from the original dataset. The median and 95% CIs (the 2.5th and 97.5th percentile values) obtained from the bootstrapping analysis were compared with those obtained from the final model.

### Simulations

Monte Carlo simulation was performed to assess ABC exposure for each WHO weight bands. An in silico pediatric population with demographics characteristic of African CLHIV was utilized [16]. Using the final population parameter estimates, the steady-state area under the curve over 24 hours (AUC_0–24_) was predicted for children receiving ABC dosed according to WHO weight band guidelines. A total of 500 000 children weighing 3–24.9 kg (100 000 children for each weight band) were simulated. The relationship between age and weight was obtained from a previous study that generated simulation data representing African CLHIV [16]. An AUC_0–24_ target between 6.4 and 50.4 mg h/L was chosen based on that observed in older children using ABC [17].

## RESULTS

### Demographic Characteristic

A total of 2254 plasma concentration data for ABC were collected from 387 African children enrolled in the LIVING study. Baseline demographic characteristics are presented in Table 1. At baseline, the average (with a range) age was 2.91 years (ranging from 0.3 to 9.7), and the body weight was 11.95 kg (ranging from 4.4 to 23).

**Table 1.** Baseline Demographic and Clinical Characteristics of Study Participants

Characteristics	Freq (%) or mean ± SD (range)
Number of subjects	387
Number of samples	2254
Gender	Male: 195 (50.39) Female: 192 (49.61)
Age in years	2.91 ± 1.74 (0.3–9.7)
Body weight in kilograms	11.95 ± 3.69 (4.4–23)
Number of children in each weigh bands	
3–5.9 kg	10
6–9.9 kg	118
10–13.9 kg	135
14–19.9 kg	116
20–24.9 kg	8
Malnutrition status: weight-for-age *z*-score (WAZ)	
Normal or mild malnutrition (WAZ > −2)	286 (73.90)
Moderate acute malnutrition (WAZ < −2 to −3)	61 (15.76)
Severe acute malnutrition (WAZ < −3)	40 (10.34)

Abbreviations: SD, standard deviation.

### Population Pharmacokinetic Analysis

A 1-compartment model with first-order absorption and elimination best described the ABC pharmacokinetics. The model fit was not improved by adding lag time and mean transit time (MTT) to account for delayed absorption. The addition of allometric scaling significantly reduced the OFV (∆OFV = −117.657). The estimation of IIV for V/F was not accurate and was not included. The inclusion of IOV on bioavailability (F) and absorption rate constant (K_a_) significantly improved the fit (∆OFV of −157.76 and −21.82, respectively).

Once the body size was adjusted through allometric scaling, a maturation effect was determined to take age-related changes in CL/F into account. The data were best characterized by the addition of the maturation effect as specified by a sigmoidal Emax model with the Hill parameter fixed to 1 (OFV = −32.39). The model estimated that clearance would be 50% of the mature value (MAT50) at 5.80 months of age, and full maturation would be reached by 5 years of age (Supplementary Figure S1). Malnutrition status was identified as a significant covariate on CL/F during forward inclusion, but it was not significant during backward deletion. The final ABC population parameters are shown in Table 2. The GOF plots did not reveal any obvious bias (Supplementary Figure S2).

**Table 2.** Final Population Pharmacokinetic Parameter Estimates of Abacavir (ABC) in Children

Parameter	Final model		Bootstrap results		
	Estimate	%RSE	2.5th	Median	97.5th
CL/F (L/h/12.9kg)	21.40	6.90	19.22	21.49	23.65
V/F (L/12.9kg)	7.20	10.80	4.83	7.02	9.56
Ka (/h)	0.37	3.60	0.33	0.36	0.40
MAT50 (years)	0.483	26.90	0.21	0.49	0.76
HILL	1 Fix	-	-	-	-
IIVCL, %CV	29.20	7.40	19.21	29.91	36.52
IIVKa, %CV	21.80	15.10	10.99	22.66	28.84
IOV F, %CV	47.60	11.20	38.73	47.01	55.23
IOV Ka, %CV	25.90	14.00	9.70	25.53	36.70
Residual variability					
Proportional (%)	51.70	4.50	47.16	52.36	55.88

Abbreviations: CL/F, apparent oral clearance; V/F, apparent volume of distribution; Ka, absorption rate constant; MAT50, postnatal age at which clearance is 50% of the mature clearance; F, bioavailability; IIV, interindividual variability; IOV, inter-occasion variability; %CV, percent coefficient variation; %RSE, relative standard error; RSE defined as: (SEestimate/estimate) × 100, where SE is standard error; RUV, residual variability.

### Model Evaluation

**Figure 1.**
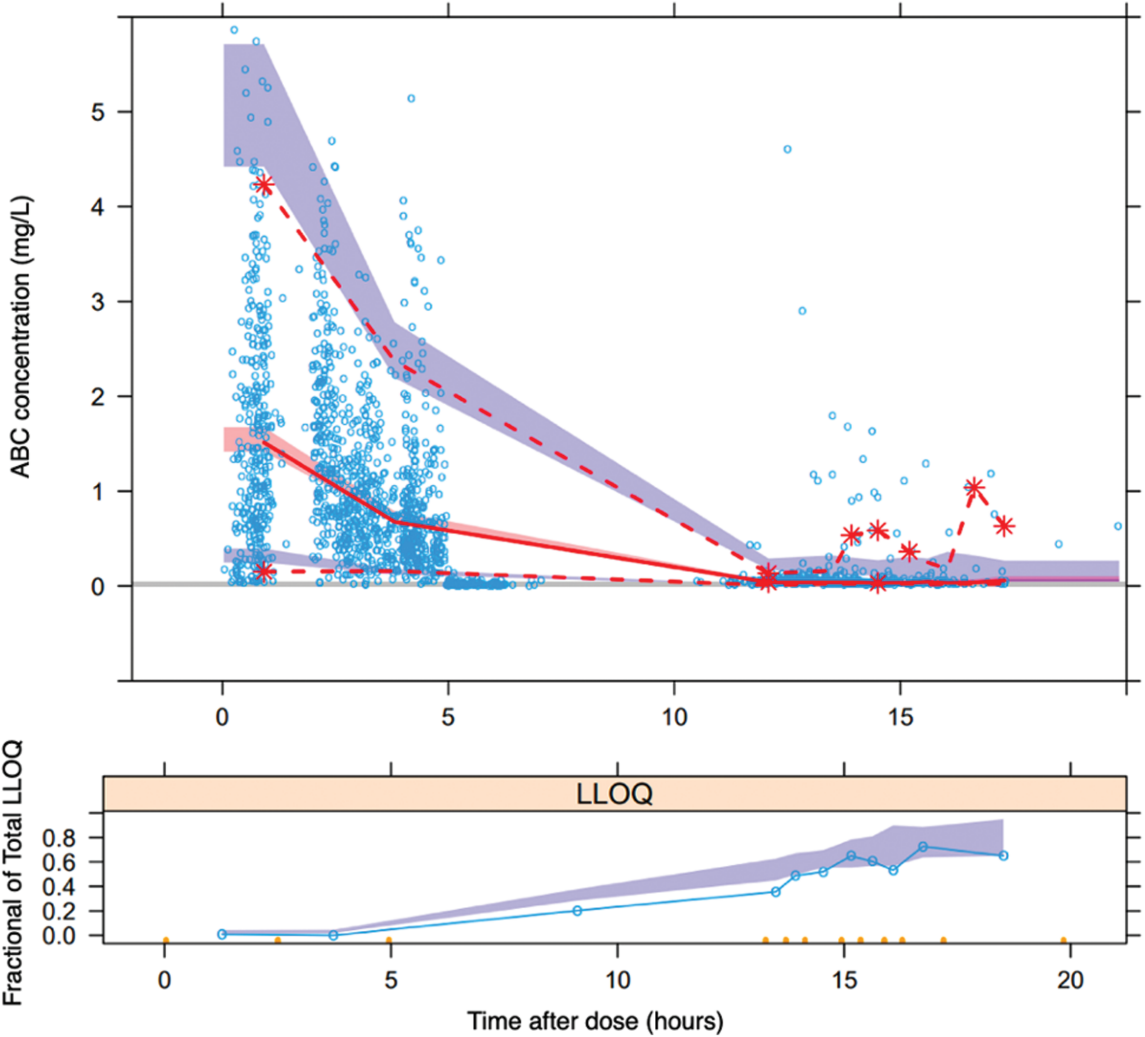
Prediction-corrected visual predictive check (pcVPC) of abacavir. Solid line is the 50th percentile, and dashed lines are the 5th and 95th percentiles of the observed concentrations, shaded areas are the 95% CIs of the corresponding model-predicted percentiles. Proportion of LLOQ values versus time after dose is shown in the bottom figure. The solid blue line represents the observed proportion of LLOQ, and the blue shaded area is the 90% confidence interval for the same proportion predicted by the model.

The pcVPC demonstrated that the 95% CIs of the relevant percentiles of the simulated data adequately represented the observed median, 5th, and 95th percentiles, as illustrated in Figure 1. 894 of the 1000 bootstrap analysis runs minimized successfully with successful covariance. The bootstrap analysis results are shown in Table 2. The bootstrap medians and their corresponding 95% CIs were comparable to the values estimated from the final model.

### Simulation for Dose Optimization

**Figure 2.**
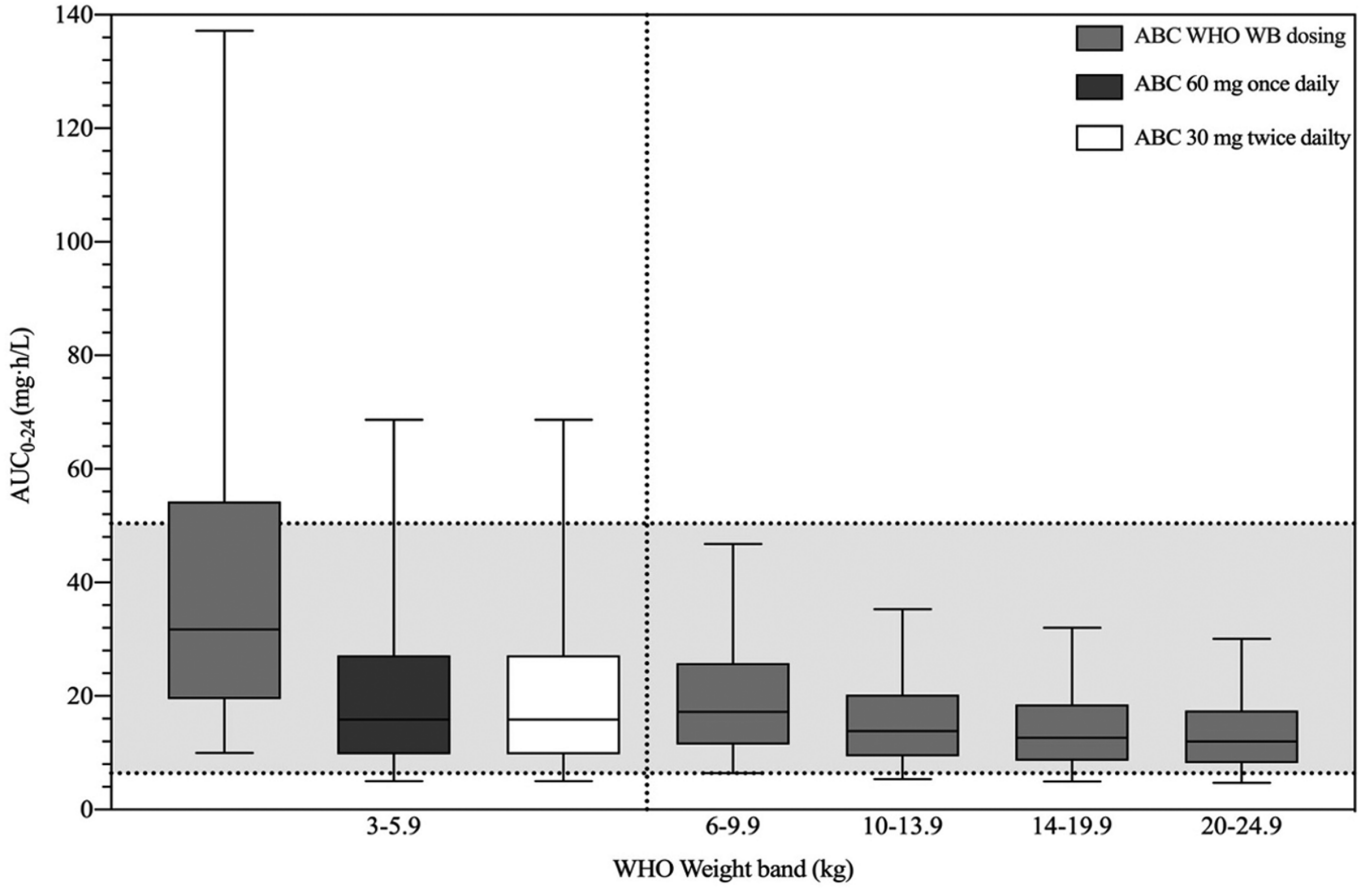
Simulated steady-state abacavir area under the curve from 0 to 24 hours (AUC_0–24_) according to WHO weight band dosing recommendations (light gray). Alternative ABC doses 60 mg OD (dark gray) and 30 mg BID (white) are also shown for the lowest weight band. Target AUC_0–24_ (6.4–50.4 mg h/L) (shaded area). The box-and-whisker plots (the lower and upper limits of individual boxes denote the 25th and 75th percentiles, and the whiskers represent 5–95th percentiles).

The ABC AUC_0–24_ per WHO weight band dosing recommendation is shown in Figure 2. The percentage of children predicted to have an AUC_0–24_ within the target range were 66%, 93%, 96%, 96%, and 95% for children 3–5.9, 6–9.9, 10–13.9, 14–19.9, and 20–24.9 kg, respectively. Thus, the majority of children achieve an ABC AUC_0–24_ within the target range, except for those in the lowest weight band. When dividing the lowest WHO weight band into the 3–3.9, 4–4.9, and 5–5.9 kg groups, the median values for ABC exposure in children weighing 3–3.9, 4–4.9, and 5–5.9 kg were 81.73, 40.18, and 24.61 mg h/L, respectively. The percentage of children with exposures within the target range was 30%, 72%, and 90%, while 0%, 0%, and 0.29% had ABC exposures below the target. Moreover, 70%, 28%, and 10% of the patients had ABC concentrations above the target. Notable, the highest variability in ABC exposures was in children 3–3.9 kg (Supplementary Figure S3). Simulation of 2 alternative ABC doses in the lowest weight band were performed, specifically 60 mg once daily, and 30 mg twice daily (compared with WHO recommended 60 mg twice daily dose for children weighing 3–5.9 kg). With a simulated doses of 60 mg once daily or 30 mg twice daily, 81% of children achieved target ABC exposures (Figure 2).

## DISCUSSION

Abacavir is a crucial component of first line antiretroviral therapy for children. Based on WHO dosing recommendations, ABC mg/kg doses with available pediatric fix dose combination tablets can be ~2.5 times higher than the approved mg/kg dose for the youngest children. The present study performed a population pharmacokinetic analysis in a large group of African infants and children to assess ABC exposures in children using a generic ABC/3TC FDC dispersible tablet dosed per WHO weight bands. Abacavir pharmacokinetics were best described by a 1-compartment model with first-order absorption and elimination, which is consistent with previous studies in children [8, 10]. Even though, a 2-compartment model has been reported for ABC in children [4, 5, 7, 9, 11], our data were relatively sparse and could not accurately identify intercompartment parameters. The population CL/F and V/F of ABC were estimated to be 21.4 L/h and 7.20 L for children weighing 12.9 kg, respectively. Our estimate of CL/F was higher than those previously reported in children weighing 12.9 kg using the liquid formulation [5–7, 9–11]. The large intersubject variability on bioavailability (F) (47.6%) and K_a_ (25.9%) observed in our study could have been due to differences in food intake. Food has been shown to impact ABC absorption, although its effect was not considered clinically important [18].

Abacavir is primarily metabolized by alcohol dehydrogenase (ADH) enzyme (30%) and uridine diphosphate glucuronosyltransferase (UGT) enzymes (36%) [19]. ADH activity is present in newborns, and by the age of 5, it reaches adult levels [20]. The influences of body weight and age were incorporated as covariates in the pharmacokinetic model to account for developmental growth and physiological changes in enzyme activity. The inclusion of weight on CL/F and V/F (allometrically scaled) significantly improved the model fit. Although part of the variability was accounted for by body weight, the addition of age describing maturation was also included. A recent population pharmacokinetic study of ABC in children with HIV/TB included both body weight and age as covariates, and the predicted maturation was almost complete by 2 years of age [21]. Our model described maturation using a sigmoidal *E*_max_ model and predicted maturation would reach 90% of adult levels by 5 years of age, which is consistent with the ADH enzyme activities reported during early life [20].

Malnutrition can cause alterations in the body’s physiology that could impact the pharmacokinetics of a drug. The influence of malnutrition status on ABC pharmacokinetics is inconclusive. Previous studies have shown that malnourished children had higher ABC exposure, perhaps due to increased bioavailability and decreased clearance [9, 11]. Reduced activity of enzymes and alterations in protein levels may also account for the high drug exposure in this population [11]. Our data indicated that neither moderate nor severe malnutrition significantly impacts ABC’s pharmacokinetics. However, our study included a limited number of children with moderate to severe malnutrition (15.76% and 10.34%, respectively). Thus, the impact of malnutrition on ABC pharmacokinetics needs to be further investigated.

Simulations to predict ABC exposure according to WHO weight band dosing showed that ABC exposures in African children with HIV were within the target exposure range reported in older children. However, the ABC exposure was found to be higher than target in the lowest WHO weight band of 3–5.9 kg with the recommended dose of ABC 60 mg twice daily. Additionally, within this weight band dosing, approximately half of the children had exposures above the target range. While a large proportion of children had exposures above target it is important to acknowledge that ABC has a good safety profile. Indeed, safety data of ABC in neonates and infants using 8 mg/kg twice daily has been shown to be safe in a large observational Cohort in South Africa [22]; however, no safety data in this population using ABC per current WHO weight band dosing [ie, 60 mg twice daily, 3 to <5.9 kg] has been reported. The simulation results showed lower doses of 60 mg once daily or 30 mg twice daily resulted in a higher percentage of children achieving ABC exposure within the target range (81% and 83%). However, as a FDC dispersible tablet of ABC/3TC was administered in our study, any proposed ABC dose reduction would also impact 3TC exposures. There is a potential risk of underdosing 3TC in this lowest weight band, particularly in infants aged >3 months, for whom the recommended dose of 3TC is 5 mg/kg twice daily [23]. Additional pharmacokinetic modeling/simulation studies are needed to determine the exposure of 3TC in the context of an ABC dose adjustment for children weighing 3–5.9 kg using an ABC/3TC FDC dispersible tablet. Despite the fact that our dose modifications may complicate pediatric regimens, the decision to adjust the ABC dosing recommendation should take into account multiple factors that balance safety risks and ease of implementation in large public health programs.

There were several limitations of our study. First, only weight, age, gender, height, and malnutrition status were investigated as potential covariates on the impact of ABC pharmacokinetics. Other patient factors, such as concurrent medication, were not available or examined in this analysis. Previously, it was shown that when ABC was combined with rifampicin, ABC exposure was lowered by 29.4%–36% [11, 21]. Children receiving rifampicin were not included in this pharmacokinetic analysis. Second, according to Anderson et al., the maturation of clearance in neonates may be characterized using either PMA or PNA. Our maturation function was based on PNA and therefore did not account for in utero maturation [24]; data on gestational age at birth was not available. Our data only included children weighing >4.4 kg, thus extrapolation for children weighing 3–4.3 kg in the lowest weight band was necessary, but the data used for simulations were representative of HIV-positive African children in terms of their demographics. ABC’s antiviral activity depends on its intracellular metabolite, carbovir. However, concentrations of carbovir were not measured in this study. A linear relationship between ABC plasma concentration and the intracellular concentration of carbovir [25] was observed in adults but its relationship in children is unknown. Finally, simulations to determine the optimal ABC dose were based on pharmacokinetic targets. Thus, the clinical implications and safety of the alternative ABC dose proposed need further investigation.

## CONCLUSION

An ABC population pharmacokinetic model was developed, and simulations support the current WHO weight band dosing, but high exposures are expected in the lowest weight band due to the high mg/kg dose. An ABC dose of 60 mg once daily or 30 mg twice daily for children weighing 3–5.9 kg is expected to achieve a high proportion of children with drug exposures within the target range.

## Supplementary Material

piad082_suppl_Supplementary_Figures_S1

piad082_suppl_Supplementary_Figures_S2

piad082_suppl_Supplementary_Figures_S3

## Notes

***Potential conflicts of interest***. E.A.B. Advisory board for MERCK, Viiv and Gilead, J.K.M. Ad-hoc advisory board for ViiV, GSK and Pfizer, V.M. Advisory board membership with ViiV Healthcare and Viatris, All other declared no conflict of interest
