# Supplementary figures and images for: Abacavir Drug Exposures in African Children Under 14 kg Using Pediatric Solid Fixed Dose Combinations According to World Health Organization Weight Bands
Source: J Pediatric Infect Dis Soc. 2023 Oct 5;12(11):574–80. doi: 10.1093/jpids/piad082 (PMC10756690; doi:10.1093/jpids/piad082)

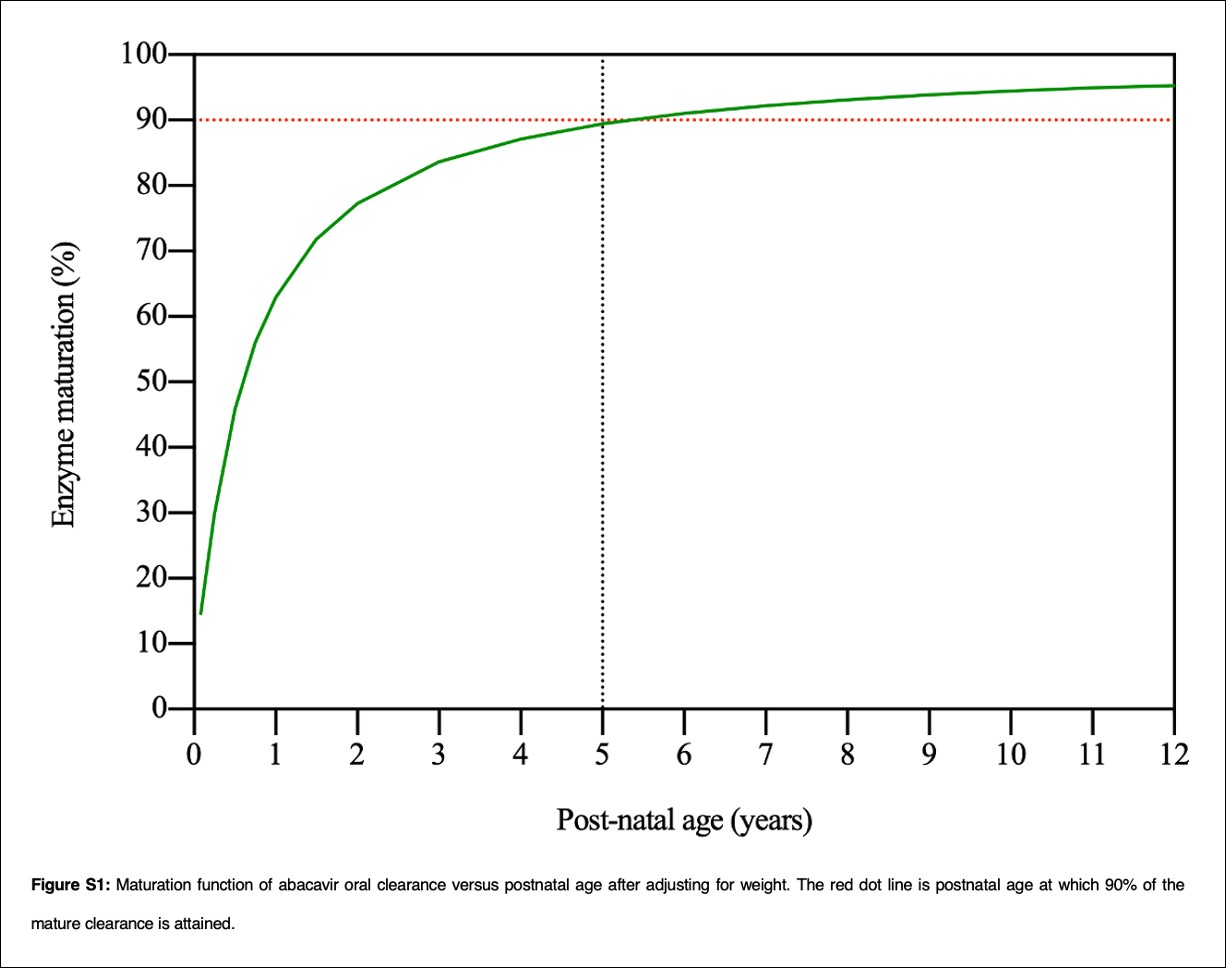

Supplement: piad082_suppl_Supplementary_Figures_S1 [file piad082_suppl_supplementary_figures_s1.jpeg]

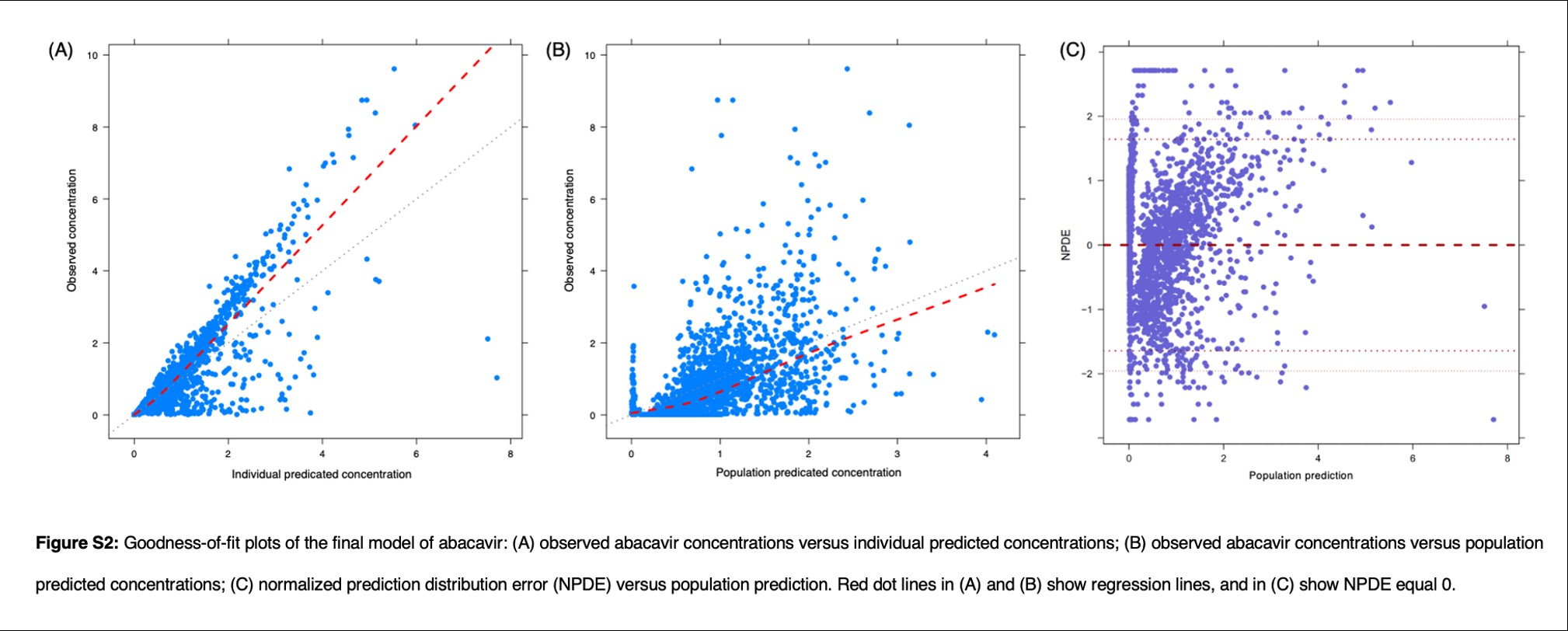

Supplement: piad082_suppl_Supplementary_Figures_S2 [file piad082_suppl_supplementary_figures_s2.jpeg]

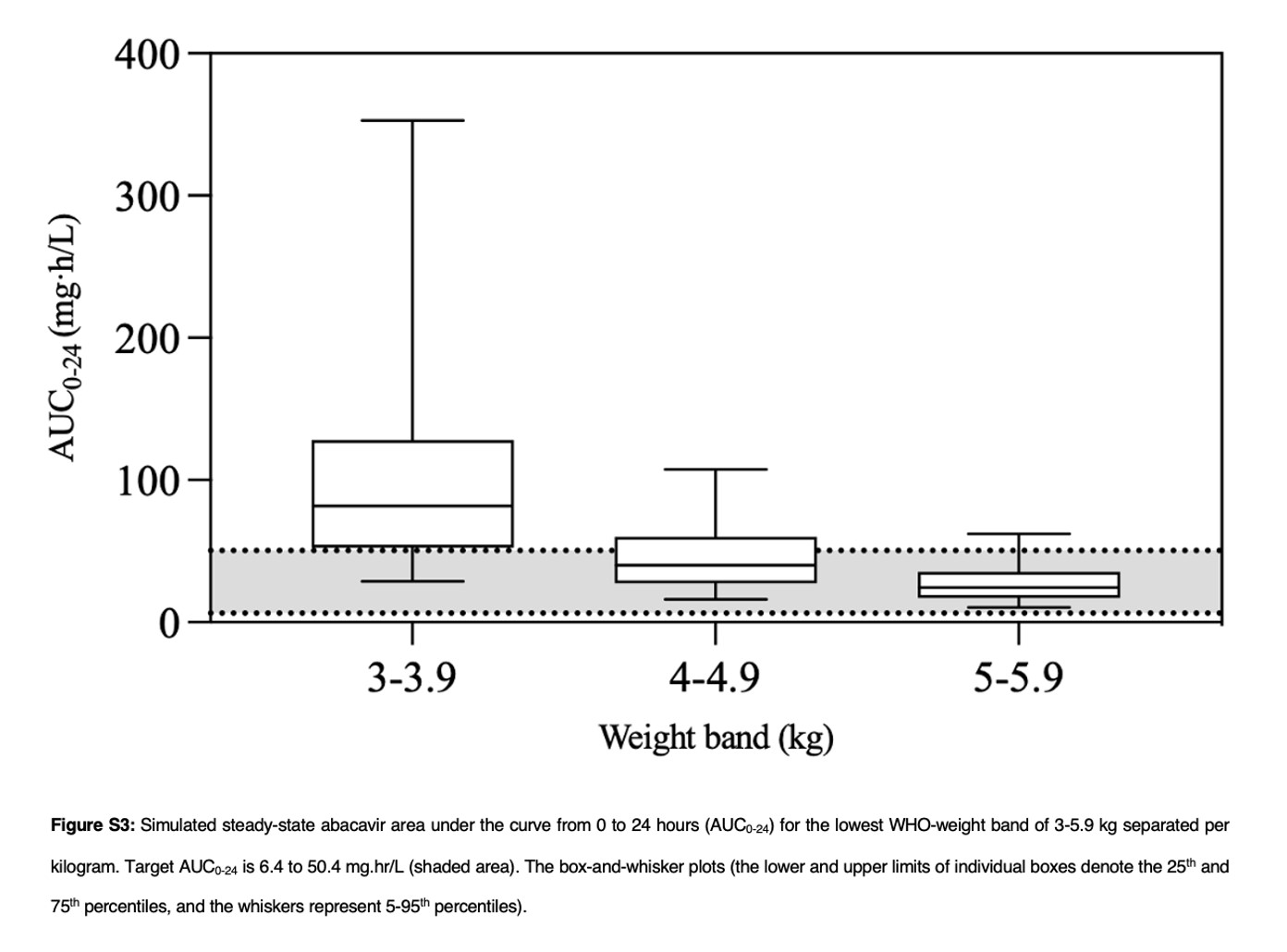

Supplement: piad082_suppl_Supplementary_Figures_S3 [file piad082_suppl_supplementary_figures_s3.jpeg]
